# Testing the Reliability and Validity of the Turkish Adaptation of Sharenting Evaluation Scale

**DOI:** 10.3390/pediatric18010009

**Published:** 2026-01-12

**Authors:** Fatih Bayraktar, Hale Ögel-Balaban

**Affiliations:** 1 Faculty of Arts & Sciences, Department of Psychology, Eastern Mediterranean University, 99628 Famagusta, North Cyprus, Cyprus; 2 Faculty of Education, University of Cambridge, Cambridge CB2 8PQ, UK

**Keywords:** sharenting, reliability, validity, scale adaptation, parents

## Abstract

**Objectives:** The aim of the present study was to adapt the Sharenting Evaluation Scale to Turkish and to test its reliability and validity. **Methods:** Through an online data collection platform, we recruited 391 parents (M_age_ = 42.2, SD_age_ = 5.6, 76% female). They have at least one child under 18 years of age and actively engage with social media. Sharenting Evaluation Scale consists of 17 items rated on a 6-point Likert scale. Its Turkish adaptation underwent a two-phase process: exploratory and confirmatory factor analyses, along with testing for construct validity. **Results:** The exploratory factor analysis revealed that the 17 items in the questionnaire were loaded onto two factors (Social Behavior and Implications). The confirmatory factor analysis indicated that the two-factor model fitted the current sample well. To evaluate the construct validity of the Sharenting Evaluation Scale, we compared groups at the extreme ends of the scoring spectrum. A *t*-test was conducted to compare the scores of both groups across subscales, revealing a significant difference. We observed positive correlations between sharenting and parental self-regulation, authoritative parenting, permissive parenting, and digital media literacy which indicate the criterion validity. **Conclusions:** In conclusion, the current study demonstrates that the Turkish version of the Sharenting Evaluation Scale is a reliable and valid tool.

## 1. Sharenting Evaluation Scale—Turkish Version

The term “sharenting,” defined as the practice of parents sharing information about their children on social media, represents a contemporary phenomenon at the intersection of parenting and digital communication [1]. Research indicates a high prevalence of sharenting in today’s digital landscape. For example, studies reveal that 98% of new mothers and 83% of new fathers in the United States, as well as 80% of parents in the Czech Republic and 90% of parents in Spain, have posted photographs of their children on social media platforms [2,3]. Similarly, Ögel-Balaban (2021) found that 81% of parents in Türkiye have shared at least one photo of their children on their own Facebook account [4]. Analysis of the content shared by parents shows that it predominantly includes photos capturing developmental milestones, daily activities such as playing or eating, special occasions like birthday celebrations, and social interactions with children [5,6,7]. Additionally, instances of parents sharing potentially embarrassing images of their children, including those depicting them in states of nudity or uncleanliness, have also been documented [5,8].

Research into the motivations behind sharenting has identified several benefits that parents derive from this online behavior. Parents often use social media to document memories and share their children’s development with their online networks. The feedback received in the form of comments and likes can provide both informational and emotional support [9,10,11,12]. Moreover, this sharing activity fosters a sense of community and connectedness, allowing parents to feel like active and supported members of a larger network [10,13]. Through their selective posting, parents also have the opportunity to curate their public image, constructing a positive representation of themselves, their children, and their family, which may enhance or repair their parental identity in accordance with societal norms [6,14,15].

Despite these advantages, sharenting poses significant risks to children. Parents inadvertently create a digital footprint for their children that includes personal details, developmental milestones, and sensitive experiences long before the children are able to manage their own online identities [5]. Furthermore, parents often share information about their children without obtaining their consent [3,6,16]. By failing to respect their children’s right to privacy, parents may also influence their children’s understanding of what constitutes private information and how it should be treated [5]. Additionally, the content shared may affect children’s future social and emotional well-being, potentially leading to feelings of shame or experiences of bullying [17,18]. Beyond privacy concerns, sharenting can jeopardize children’s security by exposing their personal information to known or unknown audiences, who may exploit this information for various harmful purposes, including marketing, financial fraud, and predatory behavior [5,18,19]. Other security risks, such as cyber-attacks and potential kidnapping, also arise from this practice [5,20].

Investigation into children’s perspectives on sharenting has revealed that they largely express concern regarding privacy risks and desire the autonomy to consent to their parents’ sharing of information [17,21,22]. Conversely, parents often exhibit a lack of concern about the risks associated with sharenting [23,24]. Moreover, studies examining the protective measures parents adopt to safeguard their children reveal that many do not adequately check the privacy and security settings of their social media accounts before sharing [6,19].

Given the widespread prevalence of sharenting and its implications for both parents and children, Romero-Rodriguez et al. (2022) highlighted the need for a tool to assess the extent of sharenting [25]. They developed the Sharenting Evaluation Scale, consisting of 17 items rated on a 6-point Likert scale, and established its reliability and validity within a sample of Spanish adults. Expert judgements on the adequacy of the items in terms of their clarity and relevance provided evidence for the content validity of the scale. The factor analysis conducted to establish its construct validity revealed three dimensions: (a) implications, which includes seven items focused primarily on the privacy and security risks associated with sharenting; (b) social behavior, consisting of six items addressing the positive and negative social feedback received by parents and children; and (c) self-control, which encompasses four items that examine the frequency with which parents share information about their children online. The overall reliability of the scale was deemed acceptable, with a Cronbach’s alpha of 0.76. The reliability of each subscale was also acceptable, with a Cronbach’s alpha of 0.87 for implications, 0.70 for social behavior and 0.67 for self-control. In general, the validity and reliability of the Sharenting Evaluation Scale were confirmed.

Sharenting has been shown to be a frequent practice among parents in Türkiye [4,7]. A well-established scale would contribute to rigorous measurement and understanding of the nature of this practice and its frequency. It could also facilitate research into its predictors and outcomes across various cultural contexts. Such insights might help bridge the gap between empirical research and practice, thereby providing valuable information to policymakers, social workers, educators, family practitioners, and families regarding the practice of sharenting. Although the Sharenting Evaluation Scale has recently been adapted for use in Türkiye by Kilic et al. (2023), this study revealed several deficiencies [26]. Notably, the study primarily relied on internet addiction as the sole variable for validating the scale, as acknowledged by the authors. Another limitation was related to its homogenous sample consisted of parents who applied to a pediatric clinic of a hospital. In addition, the scale’s factor structure was not tested via an exploratory factor analysis. These limitations necessitated further validation of the scale through exploring additional factors related to sharenting in a different sample, which we aimed to address in the present study.

We conducted an exploratory factor analysis to investigate the factor structure of the Sharenting Evaluation Scale, with the hypothesis that it would align closely with the original factor model proposed by Romero-Rodriguez et al. [25]. Given that parents often utilize posts to present and promote themselves, we anticipated a positive correlation between parental self-presentation needs and scores on the Sharenting Evaluation Scale [27]. To further validate the scale, we included parents’ self-regulation as a variable due to its relevance to the self-control subscale, which addresses parents’ oversight of their sharenting practices. Previous research has indicated a somewhat counterintuitive finding that digital literacy may not be positively associated with sharenting practices. Specifically, parents who exhibit greater comfort with social media may be more inclined to share personal content, as the perceived benefits of sharenting for themselves might outweigh the associated risks for their children [28,29]. Addressing this contentious issue, we also explored the relationship between parents’ digital literacy levels and their scores on the Sharenting Evaluation Scale. Additionally, because sharenting takes place within the context of parent–child dynamics, we incorporated parenting styles as a final variable for assessing the validity of the Sharenting Evaluation Scale [23]. Previous findings by Amon et al. (2022) suggest a positive relationship between permissive parenting styles and the frequency of sharenting, implying that permissive parents may exhibit flexibility not only in child-rearing but also in navigating issues related to their children [23]. Thus, we hypothesized a positive correlation between permissive parenting and scores on the Sharenting Evaluation Scale. On the other hand, the links between other parenting styles (i.e., authoritative/authoritarian) and sharenting are unclear. Therefore, we prefer to offer research questions instead of hypotheses at this point. Below, the summary of the hypothesis and research questions was listed:

**H1:**  *Turkish version of Sharenting Evaluation Scale is expected to be a reliable and valid measurement tool.*

**H2:**  *There will be a positive correlation between parental self-presentation needs and sharenting.*

**H3:**  *There will be a positive correlation between parental self-regulation and sharenting.*

**H4:**  *There will be a positive correlation between digital media literacy and sharenting.*

**H5:**  *There will be a positive correlation between permissive parenting and sharenting.*

RQ1: What is the association between authoritative parenting and sharenting?

RQ2: What is the association between authoritarian parenting and sharenting?

## 2. Method

### 2.1. Participants and Procedure

The data collection started after the approval of the Research and Publication Ethics Board of the Eastern Mediterranean University. G Power analysis indicated that minimum sample size should be 98 to detect a medium effect size with 80% power at a 5% significance level. We recruited a total of 391 parents (M_age_ = 42.2, SD_age_ = 5.6) through an online data collection platform (Qualtrics) with informed consent at the beginning and debriefing at the end of online survey. Participants were Turkish-speaking parents of children under 18 years of age who actively engage with social media. The study’s objectives were disseminated through social media platforms (e.g., Facebook, Twitter, Instagram) using convenience and snowball sampling methods. Data collection occurred between December 2023 and January 2024. The sample consisted predominantly of mothers (76%), most of whom reported having two children. Self-reported data indicated that 68% of participants held a bachelor’s degree or higher, and 98% maintained a social media account, with nearly half of the respondents logging in more than once daily. Reports of sharenting practices indicated a moderate frequency, with 19% to 25% of participants sharing content about their children at least once a month.

### 2.2. Instruments

**Demographic Information Questionnaire.** This questionnaire collected demographic data, including participants’ gender, age, residence, marital status (and duration of marriage), educational background, occupation, perceived socioeconomic status, and information on their children, such as age, gender, and the total number of children.

**Use of Social Media Scale.** Adapted from previous studies, this scale assessed participants’ engagement with social media. Participants answered questions regarding their primary platform, duration of use, number of friends, frequency of usage, content posting frequency, and the frequency of postings about their children [4,30].

**Sharenting Evaluation Scale.** Developed by Romero-Rodriguez et al. (2022), this scale measures the extent of sharenting among adults [25]. It consists of 17 items rated on a 6-point Likert scale ranging from 0 (never) to 5 (always), with total scores ranging from 0 to 85. The scale encompasses three dimensions: (a) implications (7 items, e.g., “How often have you felt that you were invading the minor’s privacy by sharing the child’s photograph or video?” and “How often have you considered the Child Protection Act when sharing your photo or video?”), (b) social behavior (6 items, e.g., “How often have you shared a photo or video of the minor in order to receive positive feedback from your contacts?” and “How often have you deleted the photo or video after sharing it on social media after receiving feedback from someone else?”), and (c) self-control (4 items, e.g., “How often have you shared more than one photo or video per day?” and “How often have you sent photographs or videos of the minor by private message to another person?”). The overall reliability was acceptable (Cronbach’s alpha = 0.76), with specific Cronbach’s alpha values of 0.87 for implications, 0.70 for social behavior, and 0.67 for self-control. The scale was translated into Turkish from its English version and subsequently back-translated into English by a native speaker affiliated with a university in Cyprus. Detailed reliability and validity analyses are presented below.

**Self-Presentation Subscale of Self-Focused Gratifications on Facebook Scale.** This subscale, developed by Ercan (2016), contains 60 items across five subscales [31]. The present study utilized the self-presentation subscale, comprising four items rated on a 5-point Likert scale (1 = Totally Disagree to 5 = Totally Agree). Higher average scores indicate greater self-presentation needs. The original Cronbach’s alpha for this subscale was 0.85, while it was found to be 0.82 in the current study.

**Me as a Parent Self-Regulation Scale.** Developed by Hamilton et al. (2015), this scale consists of 16 items and four subscales: self-efficacy, personal agency, self-sufficiency, and self-management [32]. Items are rated on a 5-point Likert scale (1 = Totally Disagree to 5 = Totally Agree). The original scale demonstrated good internal consistency (Cronbach’s alpha = 0.85) and was adapted into Turkish by Sarıot Ertürk (2019) [33]. In this study, the Cronbach’s alpha was found to be 0.89.

**Parental Authority Questionnaire-Revised (PAQ-R).** Developed by Reitman et al. (2002), this questionnaire encompasses three subscales: authoritarian, authoritative, and permissive parenting [34]. It consists of 30 items rated on a 5-point Likert scale (1 = Totally Disagree to 5 = Totally Agree). The Turkish adaptation was conducted by Sayıl et al. (2012) as part of a TUBITAK-SOBAG project [35]. The Cronbach’s alpha values were 0.82 for authoritative parenting, 0.81 for authoritarian parenting, and 0.56 for permissive parenting, with each subscale comprising 10 items. Internal consistencies for the subscales in the current research ranged from 0.84 to 0.91.

**Digital Literacy Scale.** Designed by Bayrakcı and Narmanlıoğlu (2021), this scale consists of 29 items across six subscales: ethics and responsibility, general information, daily use, advanced production, privacy and security, and social dimension [36]. Responses are collected using a 5-point Likert scale (1 = Totally Disagree to 5 = Totally Agree). The Digital Literacy Scale exhibited a Cronbach’s alpha of 0.93.

## 3. Results

The Turkish adaptation of the Sharenting Evaluation Scale underwent a two-phase process: exploratory and confirmatory factor analyses, along with testing for construct validity.

### 3.1. Exploratory Factor Analysis

Principal component analysis with Quartimax rotation was conducted. Quartimax rotation has been decided because it maximizes the sums of squares of the coefficients for each subscales which are also expected to be related with one structure (i.e., sharenting). The Kaiser-Meyer-Olkin (KMO) value was calculated to assess the appropriateness of the sample for factor analysis. The KMO value for the current sample was determined to be 0.87, indicating that the data was suitable for factor analysis. Also Bartlett’s test for sphericity was conducted to test whether the correlations between the items were suitable for factor analysis (χ^2^(136) = 2551.04, *p* < 0.001). The results indicated that the Exploratory Factor Analysis can be examined for the sample Following the recommendations of Cudeck and Browne (1983), the sample was split into two segments randomly; exploratory factor analysis was performed on the first segment, while confirmatory factor analysis was executed on the second [37]. The exploratory factor analysis revealed that the 17 items in the questionnaire loaded onto two factors, explaining 65.57% of the variance, with factor loadings ranging from 0.50 to 0.93. The first and second factors explained 52.87% and 12.70% of the variance, respectively (see Table 1). Internal consistency for the whole scale was 0.83. The Cronbach’s Alpha values of the first factor (e.g., Social Behavior) and second factor (e.g., Implications) were 0.86 and 0.91, respectively.

### 3.2. Confirmatory Factor Analysis

The structure of the scale was further examined through confirmatory factor analysis. The maximum likelihood estimation technique and covariance matrix were utilized to determine whether the proposed model was a good fit for the observed data. Given the sensitivity of the χ^2^ (Chi-Square) test to sample size, the sd/χ^2^ ratio criterion was used, with ratios below 1:5 considered indicative of a good fit. In accordance with the criteria established by Byrne (2001) and Hu and Bentler (1999), Comparative Fit Index (CFI), Adjusted Goodness of Fit Index (AGFI), and Non-Normed Fit Index (NNFI) values of 0.90 or above, alongside a Root Mean Square Error of Approximation (RMSEA) value of 0.08 or below, were accepted as reflecting a good fit between the proposed model and the data [38,39].

**Figure 1:**
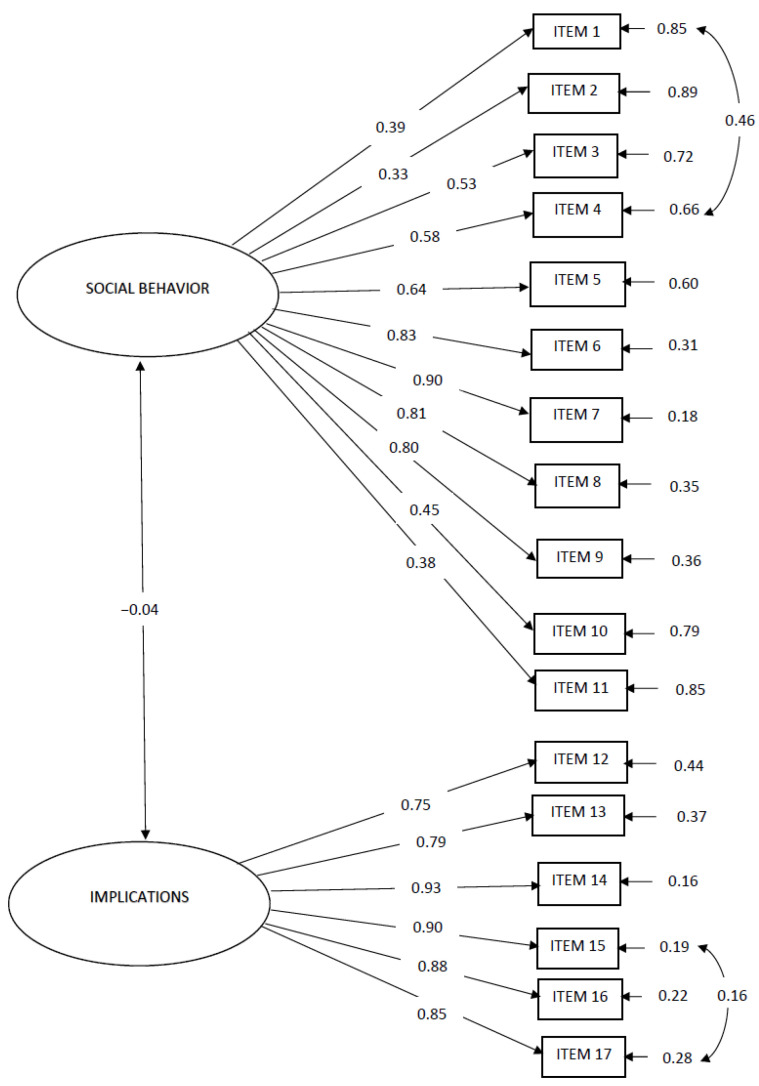
Confirmatory Factor Analysis of Sharenting Evaluation Scale—Turkish Version.

Modification indices suggested enhancing the model by correlating the errors between Item 1 (How often have you shared pictures or videos of the minor on your social media profile?) and Item 4 (How often have you felt the need to share the minor’s photographs or videos on social media?), as well as between Item 16 (How often have you considered that the photographs or videos you have shared of the minor could be used for identity theft on the Internet?) and Item 17 (How often have you considered that the photographs or videos you have shared of the minor could end up on websites that promote pedophilia?). It was posited that these items may be perceived similarly by parents, warranting the correlation of their errors (standardized error coefficients = 0.16 and 0.46, respectively). Following these modifications, the confirmatory factor analysis indicated that the two-factor model fitted the current sample well, [χ^2^ (116, N = 391) = 534.64, *p* < 0.001, RMSEA = 0.08, GFI = 0.90, AGFI = 0.90, CFI = 0.94, NNFI = 0.93]. Figure 1 illustrates the standardized coefficients among the indicator variables, latent variables, and the error variances of the indicator variables; all coefficients are significant at the *p* < 0.001 level, except for the standardized coefficient between the latent variables (i.e., Social Behavior and Implications).

### 3.3. Construct Validity

To evaluate the construct validity of the Sharenting Evaluation Scale, we compared groups at the extreme ends of the scoring spectrum. The top 27% of the sample was designated as the upper group, while the lowest 27% was named the lower group. A *t*-test was conducted to compare the scores of both groups across subscales, revealing a significant difference (*t* = −19.85, *p* < 0.001). Group means, standard deviations, and *t*-test results are presented in Table 2.

We also examined the Pearson correlation coefficients between sharenting and other measured constructs. The observed correlations partially aligned with our expectations, as demonstrated in Table 3.

## 4. Discussion

The present study sought to evaluate the psychometric properties of the Sharenting Evaluation Scale within a Turkish context [25]. Although a previous adaptation of the scale was attempted by Kilic et al. (2023), several limitations were identified [26]. First, the sampling method raised concerns, as participants were recruited solely from parents visiting a pediatric outpatient clinic, leading to a relatively homogenous sample as noted by the authors. Second, no exploratory factor analysis was conducted prior to confirmatory factor analysis to test the scale’s factor structure. Third, while criterion validity was assessed, it was limited to a single variable (i.e., internet addiction) without exploring construct validity comprehensively. To address these shortcomings, our study aimed to recruit a more heterogeneous sample and conduct a thorough analysis.

The exploratory factor analysis indicated that items clustered under two dimensions—Social Behavior and Implications. Notably, all items originally categorized under Self-Control in the initial study were found to align with Social Behavior in our analysis. This may suggest that parents in our sample exhibit “oversharenting” alongside regular sharenting, indicating that sharenting occurs along a continuum. This structure was corroborated by confirmatory factor analysis. In contrast to the original study, the latent variables of Implications and Social Behavior exhibited a negative correlation that was not statistically significant. This suggests that practices aimed at protecting children and those related to sharenting may not overlap within our sample, implying the utility of distinguishing these two dimensions, which reflect different motivations for sharenting.

The total score of sharenting can also represent the broader spectrum of this behavior. Among the Pearson correlation coefficients calculated between the total sharenting score and other variables, the results were partially as anticipated. Our selected variables were grounded in existing literature. We observed positive correlations between sharenting and parental self-regulation, authoritative parenting, permissive parenting, and digital media literacy. The correlation involving digital media literacy contradicted some prior findings, indicating the controversy surrounding the relationship between these constructs [28,29]. Such discrepancies may arise from variations in measuring sharenting. Employing quantitative methods with consistent measurement tools may help mitigate conflicting findings. The correlation between sharenting and permissive parenting was consistent with previous studies [23]. Furthermore, we identified a positive relationship between sharenting and authoritative parenting, highlighting that both parenting styles—authoritative and permissive—share a responsive, nurturing, and involved approach [40]. The dynamics of authoritarian parenting differ significantly, characterized by psychological distance and lack of involvement [41].

The anticipated link between parental self-regulation and sharenting was also supported by our findings. This suggests that parents in our sample perceive themselves as competent in self-regulation, indicating that their sharenting practices are generally well-controlled. This perception may explain the non-significant relationship observed between parental self-presentation and sharenting; specifically, parents may lack motivation to present or promote themselves through sharenting due to their controlled practices.

The present study has several limitations. First, the sample consisted of mostly participants with a university degree which limited the generalizability of the findings. Another limitation was that, although the Sharenting Evaluation Scale was developed for the Spanish population, for the present study, its items were translated from their English version which were not validated. Lastly, data was based on self-reports. The parents could give biased responses especially for risky parental practices.

In conclusion, the current study demonstrates that the Turkish version of the Sharenting Evaluation Scale (SES-T) is a reliable and valid tool. Similarly to the global context, there has been a notable gap in quantitatively measuring sharenting in Türkiye and Northern Cyprus, representing a significant barrier to understanding sharenting dynamics. Through the use of SES-T, Turkish-speaking researchers will be equipped to assess both the protective and risk factors associated with sharenting in a more comprehensive manner. Additionally, the scale can serve practitioners (e.g., social workers, psychologists, counselors) striving to protect children from the potential misuse of digital media by their parents.

## Figures and Tables

**Table 1:** Results from the Exploratory Factor Analysis of the Sharenting Evaluation Scale-Turkish Version.

Item	Factor Loading	
	1	2
Factor 1: Social Behavior		
1. How often have you shared pictures or videos of the minor on your social media profile?	0.60	
2. How often have you sent photographs or videos of the minor by private message to another person?	0.50	
3. How often have you shared more than one photo or video per day?	0.61	
4. How often have you felt the need to want to share the minor’s photographs or videos on social media?	0.72	
5. How often have you shared a photo or video of the minor in order to receive positive feedback from your contacts?	0.77	
6. How often have you shared photographs or videos of the minor in intimate situations (e.g., nude or seminude, in swimwear or in situations where sensitive information is exposed)?	0.73	
7. How often have you shared photographs or videos that may cause frustration and/or embarrassment to the minor?	0.76	
8. How often have you shared pictures or videos of other minors that you have received from other people (e.g., pictures of children of a family member or friend or even memes, stickers or viral videos)?	0.74	
9. How often have people around you reproached you for sharing photos or videos of the minor?	0.71	
10. How often have you deleted the photo or video after sharing it on social media after receiving feedback from someone else?	0.57	
11. How often have you felt that you were invading the minor’s privacy by sharing the child’s photograph or video?	0.51	
Factor 2: Implications		
12. How often have you considered the Child Protection Act when sharing your photo or video?		0.80
13. How often have you considered that the photographs or videos you share on social media are creating a digital footprint of the minor?		0.83
14. How often have you considered that the photograph or video shared may have a negative impact on the minor’s future?		0.92
15. How often have you considered that sharing a photo or video presents a risk to the minor?		0.90
16. How often have you considered that the photographs or videos you have shared of the minor could be used for identity theft on the Internet?		0.93
17. How often have you considered that the photographs or videos you have shared of the minor could end up on websites that promote paedophilia?		0.90

**Table 2:** Means (Standard Deviations) of Upper and Lower Groups for Sharenting Evaluation Scale- Turkish Version.

Variable	*Lower Group* *(n = 107)*	*Upper Group* *(n = 112)*	*t*
**Sharenting score**	**31.07 (7.71)**	**52.45 (7.62)**	−19.84 ***

*** *p* < 0.001.

**Table 3:** Descriptive Statistics and Correlations for Variables.

	*M*	*SD*	1	2	3	4	5	6	7
1. Sharenting	47.98	16.70	_						
2. Self presentation	26.11	6.72	−0.04	_					
3. Parental Self-regulation	199.71	23.59	0.24 **	−0.20 **	_				
4. Authoritative parenting	25.23	2.50	0.31 **	−0.01	0.69 **	_			
5. Authoritarian parenting	33.65	6.28	0.04	0.25 **	−0.30 **	−0.18 **	_		
6. Permissive parenting	28.79	4.51	0.12 *	0.25 **	0.04	0.14 **	0.05	_	
7. Digital media literacy	23.38	3.06	0.13 *	−0.04	0.29 **	0.33 **	−0.08	0.07	_

* *p* < 0.05, ** *p* < 0.001.

## Data Availability

The authors declare that the data is available and can be sent to the editors if asked.
